# Study on the Polarization of Astrocytes in the Optic Nerve Head of Rats Under High Intraocular Pressure: In Vitro

**DOI:** 10.3390/bioengineering12020104

**Published:** 2025-01-23

**Authors:** Bochao Ma, Jifeng Ren, Xiuqing Qian

**Affiliations:** School of Biomedical Engineering, Capital Medical University, Beijing 100069, China; mabochao@mail.ccmu.edu.cn (B.M.);

**Keywords:** glaucoma, astrocyte polarization, high intraocular pressure, optic nerve head astrocytes

## Abstract

Astrocytes, the most common glial cells in the optic nerve head (ONH), provide support and nutrition to retinal ganglion cells. This study aims to investigate the polarization types of astrocytes in the ONH of rats under high intraocular pressure (IOP) and explore signaling pathways potentially associated with different types of polarized astrocytes. The rat models with chronic high IOP were established. High IOP lasted for 2, 4, 6, and 8 weeks. Astrocytes were extracted from the ONH of rats using the tissue block cultivation method. Western blot was used to detect the expression of proteins associated with astrocyte polarization. Proteomics was employed to identify differential proteins associated with astrocyte polarization. Astrocytes polarized into A2 astrocytes after 2, 4, 6, and 8 weeks of high IOP, while polarization into A1 astrocytes began only after 8 weeks of high IOP. The differential proteins associated with A1 astrocyte polarization are primarily enriched in pathways of neurodegeneration with respect to multiple diseases, while the differential proteins associated with A2 astrocyte polarization are primarily enriched in pathways of spliceosome in amyotrophic lateral sclerosis. Our findings could provide a better understanding of the role of ONH astrocytes in the pathogenesis of glaucoma and offer new perspectives for glaucoma treatment.

## 1. Introduction

Glaucoma is an irreversible ocular disorder causing blindness and is the world’s second most common cause of blindness, following cataracts [1]. Over 60 million individuals globally had glaucoma in 2019, with projections estimating nearly 95.4 million cases by 2030 [2]. Although the cause of glaucoma remains mysterious, an elevated intraocular pressure (IOP) is certainly considered to be a key contributing factor [3]. The optic nerve head (ONH) is a critical site of early glaucomatous damage, characterized by significant changes in tissue morphology, disruption of axonal transport, and severe axonal injury [4,5,6,7]. The lamina cribrosa (LC), a connective tissue structure located in the ONH, provides support for the retinal ganglion cell axons as they exit the eye wall [8]. Astrocytes are the predominant glial cells in ONH and have a supportive, nutritious function for retinal ganglion cells (RGCs) [9,10].

Astrocytes stay in a resting state under normal conditions and change to a more reactive and proliferative phenotype after injury or disease, a phenomenon which is called astrocyte activation [11,12]. Studies have shown that activation of ONH astrocytes is among the earliest responses to increased IOP and is frequently detected before any visible axon damage [13]. Researchers have found that astrocytes are activated by an increase in IOP. When subjected to persistently elevated IOP, astrocytes undergo significant structural transformations. These changes include a noticeable thickening of both the cell body and its extensions, a retraction and realignment of the primary processes, and a marked increase in the number of both primary and secondary branches [14,15,16]. Besides morphological alterations, reactive astrocytes increase the expression of “Pan-reactive” molecules such as glial fibrillary acidic protein (GFAP), vimentin, and lipocalin-2 [17]. Furthermore, astrocytes respond to significant biomechanical deformations in the ONH through reactive astrocyte gliosis [18,19].

Analysis of transcriptomic data reveals that reactive astrocytes are categorized into two subtypes: A1 and A2 phenotypes [20,21]. A1 astrocytes are primarily activated by interleukin-1α (IL-1α), tumor necrosis factor-α (TNF-α), and complement component 1q (C1q) released by microglia in neuroinflammatory models [21]. Mitochondrial fragments released by microglia, or astrocytes treated with interleukin-18 (IL-18) in vitro, can induce astrocytes to polarize into the A1 phenotype, which is detrimental to neurons [22,23]. Studies have shown that the conditioned medium derived from human reactive A1-like astrocytes exhibits neurotoxicity toward both human and rodent neurons [24]. A1 astrocytes secrete neurotoxic factors such as complement protein C3 and inflammatory cytokines, exerting direct or indirect toxic effects on RGCs [21]. These factors may accelerate RGC apoptosis and degeneration. In addition to proteins, saturated lipids present in APOE and APOJ lipoparticles are also responsible for mediating astrocyte-induced toxicity [25]. Studies have shown that astrocytes promote central nervous system axon regeneration by forming glial scars [26]. Further studies indicate that the polarization of astrocytes into A2 astrocytes enhances synapse formation following acute ischemic spinal cord injury by regulating Glypican 6 via the STAT3 signaling pathway [27]. In addition, A2 astrocytes enhance neuronal survival and tissue regeneration through the secretion of neuroprotective factors like the transforming growth factor-β (TGF-β), thrombospondins, the brain-derived neurotrophic factor (BDNF), the glial-cell-line-derived neurotrophic factor (GDNF), and the nerve growth factor (NGF) [28,29,30]. Diminishing A1 astrocyte polarization or enhancing A2 astrocyte polarization exerts neuroprotective benefits [31,32,33]. Research on astrocyte polarization has mainly concentrated on neurodegenerative disorders, injuries, and infections [18,34]. Research has found that astrocytes become activated under chronic IOP. Therefore, studying the polarization of astrocytes in the ONH of rats under chronic high IOP will provide new insights into the treatment of glaucoma.

This paper establishes a chronic high IOP rat model for 2, 4, 6, and 8 weeks and extracts astrocytes from the ONH. The expression of proteins associated with astrocyte polarization is detected using a western blot and differential proteins associated with astrocyte polarization are identified using proteomics. Finally, we explore signaling pathways potentially associated with different polarized astrocyte types which could offer a crucial foundation for glaucoma diagnosis and treatment.

## 2. Materials and Methods

### 2.1. Animals

Twenty male Sprague Dawley (SD) rats, aged 7–8 weeks and weighing between 270 and 300 g, were obtained from the Experimental Animal Department at the Capital Medical University. They were given a minimum of 24 h to acclimate. They were kept in an air-conditioned room with a 12 h light/dark cycle, having free access to food and water. All rats were randomly divided into five groups: a single control group and four experimental groups, which were observed 2 weeks, 4 weeks, 6 weeks, and 8 weeks after creating a model of sustained elevated intraocular pressure (IOP). The control group contained normal SD rats aged 7–8 weeks that were not subjected to the high IOP model induction. Before the experiment, each rat was screened to ensure their corneas were intact, the intraocular pressure was normal, and they had no other ocular diseases. All animal experiments complied with the guidelines for animal care described in the Statement for Use of Animals in Ophthalmic and Vision Research by the Association for Research in Vision and Ophthalmology.

### 2.2. Model Induction and IOP Measurement

The chronic high IOP model in rats was established through episcleral vein cauterization and by administering a subconjunctival injection of 5-fluorouracil (5-Fu, Haipu Pharmaceutical Co., Shanghai, China) following established research protocols [35,36]. Rats were anesthetized intraperitoneally with 0.4 mL of 1% sodium pentobarbital (Sigma-Aldrich, St. Louis, MO, USA) per 100 g of body weight. Deep anesthesia was confirmed by the absence of a pain response and pupillary reflex. For the high IOP experimental group, surface anesthesia of the cornea and periocular tissues was achieved using oxybuprocaine hydrochloride eye drops (Santen Pharmaceutical, Osaka, Japan) in the right eye. A high-temperature electrocoagulation pen was used to cauterize three to four upper scleral vein trunks in the right eye. Successful cauterization was indicated by the marked discoloration and whitening of the upper scleral vein distal to the wound due to the blocked blood flow and the congestion and swelling of the vein near the wound. A 100 μL subconjunctival injection of 25 mg/mL 5-Fu was given with a 29G needle to suppress neovascularization. Levofloxacin eye drops (Santen, Osaka, Japan) were applied to the ocular surface to prevent inflammation.

IOP was measured in unanesthetized rats every three days using a TonoLab tonometer (iCare, Vantaa, Finland). To minimize circadian rhythm effects, measurements were recorded from 10 a.m. to 12 a.m. Following induction, IOP measurements continued every three days. If IOP was found to be below 30 mmHg, additional cauterization was performed. If IOP was 30 mmHg or higher, only a 5-Fu injection was administered to prevent the development of new blood vessels.

### 2.3. Isolation of Astrocytes from Adult Rat Lamina Cribrosa

Adult SD rats received an intraperitoneal injection of 10% chloral hydrate (Leagene, Beijing, China) (4 mL/kg). After the rats were anesthetized, the eyeballs were extracted and rinsed two to three times using sterile phosphate-buffered saline (PBS) containing 0.1% gentamycin (Sigma-Aldrich, St. Louis, MO, USA). The eyes were then placed in a sterile culture dish, and all subsequent operations were performed under sterile conditions. Under a dissecting microscope, the sheath, blood vessels, and fibrous tissues surrounding the ONH were removed. The ONH region and a small segment of the optic nerve behind it were isolated and cut into five to eight tissue blocks. The tissue blocks were washed two to three times with PBS and then placed into culture dishes containing standard culture medium (DMEM/F-12 (Gibco, Waltham, MA USA) with 10% fetal bovine serum (FBS, Gibco, Waltham, MA USA) and 1% penicillin/streptomycin (Hyclone, Logan, UT, USA) for adherent growth. The culture dishes were incubated at 37 °C in a 5% CO_2_ incubator, with the medium replaced every two days. After approximately 15 days, astrocytes migrated out from the tissue blocks.

### 2.4. Cell Passaging and Purification

When the cells grew to approximately 70% of the culture dish, the tissue blocks were removed, and the cells were treated with 2.5 g/L trypsin (Gibco, Waltham, MA, USA) for 2 min to create a single-cell suspension. This suspension was transferred to a primary glial cell culture medium (with a composition ratio of 100:10:1:1 for basal medium, FBS, primary glial cell supplements, and penicillin/streptomycin, respectively; PriCells, Jiangyin, China) for selective cell culture through two passages. The cultures were incubated in an incubator, with the medium replaced every two days.

### 2.5. Cell Immunofluorescence Staining

Cells were fixed at room temperature for 10 min using 4% paraformaldehyde (Beyotime, Nantong, China). Subsequently, they were permeabilized for 10 min with 0.1% Triton X-100 in PBS (Beyotime, Nantong, China). After a 2 h blockage with 5% BSA (Solarbio, Beijing, China) at room temperature, the cells were incubated overnight in the dark at 4 °C with the primary antibodies listed below: rabbit polyclonal to GFAP (RRID: AB_305808, 1:1000, Abcam, Cambridge, UK) and vimentin (RRID: AB_10695459, 1:500, CST, Danvers, MA, USA). After washing with PBS three times, the cells were incubated with 488-conjugated goat polyclonal secondary antibody to rabbit IgG(H+L) (RRID: AB_956012, 1:500, Abcam, Cambridge, MA, USA) for 2 h at 37 °C in the dark. The cells were incubated with DAPI (4′,6-diamidino-2-phenylindole, Abcam, Cambridge, MA, USA) for 5 min, then washed twice with PBS, each time for 5 min, and observed under a fluorescence microscope (Axio Observer D1, Zeiss, Oberkochen, Germany).

### 2.6. Protein Extraction and Digestion

Cells were removed from the incubator and washed twice using PBS washes, each time for one minute. The RIPA lysis buffer (Thermo Scientific, Waltham, MA, USA), at a ratio of 200 µL per 5 × 10^6^ cells, was then added, and the cells were disrupted on ice for 30 min. Following centrifugation, the supernatant was obtained as the protein extract. Then, quantitative electrophoresis was carried out, and the protein extract concentration was determined using the Bradford assay [37]. To confirm the purity of the extracted proteins, 10 μg of samples was subjected to separation via 12% SDS-PAGE. For enzymatic digestion, each sample containing 100 μg of protein was treated with 2.5 μg of trypsin (maintaining a protein-to-enzyme ratio of 40:1) for 4 h at 37 °C. The enzymatic peptides were subsequently desalted via a Strata X column, vacuum-dried, and prepared for mass spectrometry analysis.

### 2.7. Data-Dependent Acquisition (DDA) and Data-Independent Acquisition (DIA) Analysis by Nanoscale Liquid Chromatography Coupled with Tandem Mass Spectrometry

Proteomic analyses were conducted with an UltiMate 3000 UHPLC system (Thermo Fisher Scientific, Waltham, MA, USA). Following the protocol established in previous studies [38,39], the dried peptide samples were dissolved in a buffer with mobile phase A (2% acetonitrile, Sigma-Aldrich, St. Louis, MO, USA; 0.1% formic acid *v*/*v*, Sigma-Aldrich, St. Louis, MO, USA). The samples were centrifuged at 20,000× *g* for 10 min at 4 °C, followed by enrichment and desalting of the supernatant using a trap column. The sample supernatant was loaded onto a tandem column with an internal diameter of 150 μm, a particle size of 1.8 μm, and a length of 35 cm. Separation was carried out at a flow rate of 500 nL/min, employing a gradient protocol that began with 5% of mobile phase B (a mixture of 98% acetonitrile and 0.1% formic acid, *v*/*v*) for the first five minutes. From 5 to 120 min, the concentration of mobile phase B was gradually increased from 5% to 25%. It was then increased from 25% to 35% between 120 and 160 min, followed by an increase from 35% to 80% from 160 to 170 min, maintained at 80% of mobile phase B from 170 to 175 min, and, finally, returned to 5% of mobile phase B from 175 to 180 min.

For the DDA analysis, peptides separated by LC were ionized using a nano-electrospray and introduced into a tandem MS Q-Exactive HF X (Thermo Fisher Scientific, Waltham, MA, USA) operating in DDA detection mode. The MS parameters were configured with the following settings: an ion source voltage of 1.9 kV, a scan range spanning 350 to 1500 *m*/*z*, a resolution of 120,000, a maximum injection time (MIT) capped at 100 ms, and an automatic gain control (AGC) target set to 3 × 10^6^. These specifications were carefully chosen to optimize the instrument’s performance and ensure precise data acquisition. The high-energy collision-induced dissociation (HCD) parameters, integrated with tandem mass spectrometry, were configured as follows: 28 normalized collision energy levels, a resolution of 30,000, a maximum injection time (MIT) of 100 ms, an AGC target of 1 × 10^5^, a dynamic exclusion window of 30 s, and an isolation window set to 2.0 *m*/*z*. For the DIA analysis, the same nano-liquid chromatography (nano-LC) system and gradient used in the DDA analysis were applied. Critical parameters included an MS scan range of 400–1250 *m*/*z*, a resolution of 120,000, an MIT of 50 ms, an AGC target of 3 × 10^6^, and 45 loop counts across the 400–1250 *m*/*z* range. In the high collision energy dissociation mode paired with tandem mass spectrometry, fragment ions were analyzed in the orbitrap at a resolution of 30,000, with automatic MIT, an AGC target of 1 × 10^6^, 45 loop counts, a dynamic exclusion period of 30 s, and stepped normalized collision energies of 22.5, 25, and 27.5.

### 2.8. DIA-MS Data Analysis

The spectral library was generated from MaxQuant (v.1.5.3.30) DDA search outcomes utilizing Spectronaut with standard configurations [40]. Using the existing spectral library, the DIA data were processed and analyzed with Spectronaut (12.0.20491.14.21367) [41]. The study was conducted using a dynamic indexed retention time prediction model, with decoy sequences generated through scrambling. A stringent Q-value threshold of 1% was enforced for both peptide and protein identification. Local normalization was employed as the primary strategy for data standardization. Precursor-level data extracted from the Spectronaut standard report was subsequently analyzed and refined using the MSstats software for advanced processing [42]. This process involved applying a log transformation to the intensity values, performing median normalization across various conditions, and summarizing the protein data. To assess differentially expressed proteins (DEPs) according to the established comparison groups, we employed the mixed linear model available in the MSstats R package (v.3.2.1, http://msstats.org, accessed on 28 October 2015) [42]. The identification of peak regions was accomplished by utilizing a DIA secondary fragment ion, necessitating at least one distinct peptide along with a minimum of three transitions for each peptide. In the bioinformatics analysis, the proteins that were identified were annotated and categorized into pathways by referencing the Gene Ontology (GO) database (2018 version) and the Kyoto Encyclopedia of Genes and Genomes (KEGG) database (2019 version). Statistically significant differences were selected based on a fold change >2 and *p* < 0.05.

### 2.9. Western Blotting

Cells were harvested and lysed in RIPA lysis buffer (Thermo Scientific, Waltham, MA, USA), at a ratio of 200 µL per 5 × 10^6^ cells, containing a protease inhibitor cocktail (Millipore, Temecula, CA, USA). The protein extracts were resolved using SDS–PAGE and then transferred to PVDF membranes (Sigma-Aldrich, St. Louis, MO, USA). To prevent nonspecific binding, the membranes were treated with a 5% nonfat milk solution and left to sit at room temperature for a couple of hours. Afterward, they were transferred to a cold environment at 4 °C and allowed to incubate overnight in the presence of the specified primary antibodies: anti-GAPDH (RRID:AB_2107436, 1:1000, Proteintech, Rosemont, IL, USA), anti-GFAP (RRID:AB_305808, 1:1000, Abcam, Cambridge, UK), anti-ATP1B1 (RRID:AB_2290040, 1:2000, Proteintech, Rosemont, IL, USA), anti-HIP1R (RRID:AB_2117572, 1:2000, Proteintech, Rosemont, IL, USA), anti-WDR4 (RRID:AB_3674305, 1:5000, Solarbio, Beijing, China), anti-C3 (RRID:AB_2924273, 1:1000, Abcam, Cambridge, UK), and anti-S100A10 (RRID:AB_2269906, 1:1000, Proteintech, Rosemont, IL, USA). All antibodies were diluted with 5% nonfat milk. Following this, the membranes were incubated with HRP-conjugated goat anti-rabbit IgG(H+L) secondary antibody (RRID: AB_2313567, 1:1000, Jackson ImmunoResearch Labs, West Grove, PA, USA) for 1 h at 37 °C. The relative intensities of the bands were quantified using Image Lab (Bio-Rad, Hercules, CA, USA) with enhanced chemiluminescence detection (Millipore, Temecula, CA, USA).

### 2.10. Messenger RNA Extraction and qPCR

Total RNA was isolated with the TRIZOL reagent (Thermo Scientific, Waltham, MA USA), and 2 μg of RNA was utilized for the synthesis of complementary DNA (cDNA). The expression levels of mRNA corresponding to differential proteins were measured using SYBR Green (Bio-Rad, Hercules, CA, USA) and analyzed on the CFX96 Real-Time System (Bio-Rad, Hercules, CA, USA).

### 2.11. Statistical Analyses

The data are presented as the mean ± standard deviation. One-way analysis of variance (ANOVA) was used to compare data among multiple groups. The Bonferroni correction was applied following the one-way ANOVA for multiple comparisons. All analyses were conducted using SPSS 26.0 (IBM Corporation, Armonk, New York, NY, USA), with a *p*-value of less than 0.05 regarded as statistically significant.

## 3. Results

**Figure 1:**
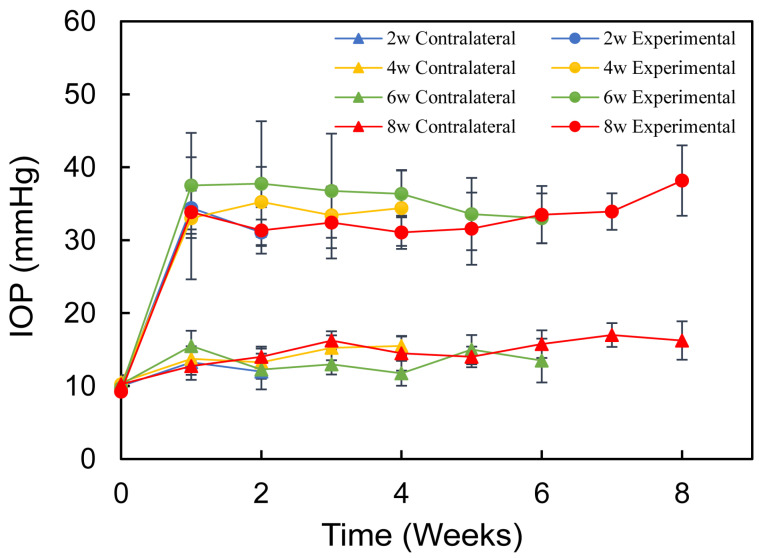
IOPs in the experimental eyes showed a significant increase following model induction (*p* < 0.05), whereas the IOPs in the contralateral control eyes stayed within normal limits (*p* > 0.05).

The IOPs of the rats’ eyes were recorded while awake prior to the experiment. After establishing the chronic high IOP model of rat, IOPs in the experimental eyes increased and remained elevated for 8 weeks, while the contralateral eyes retained normal IOP. This indicated that the chronic high IOP model of rat was successfully established (Figure 1).

**Figure 2:**
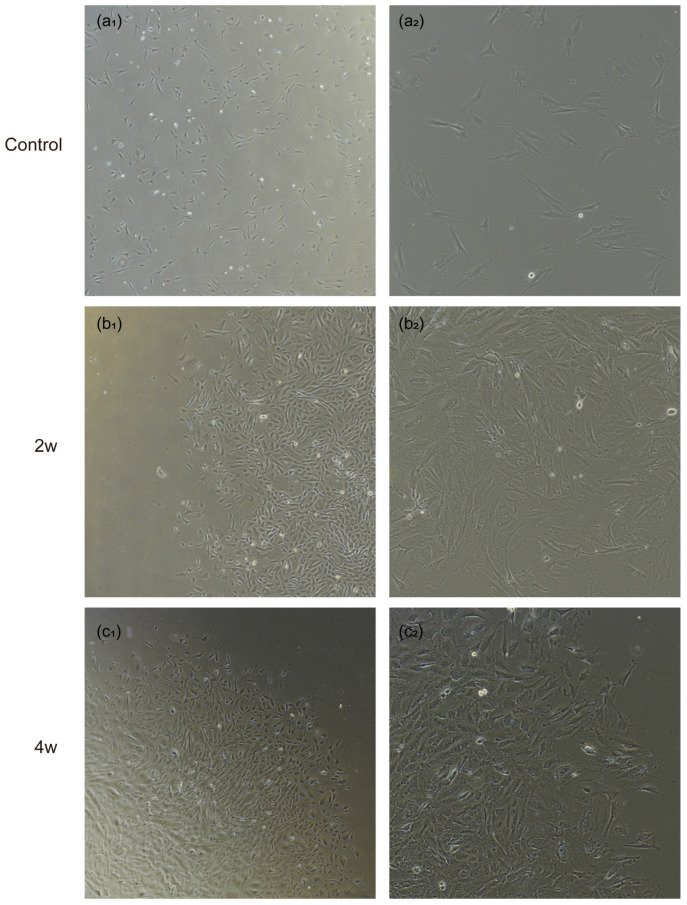

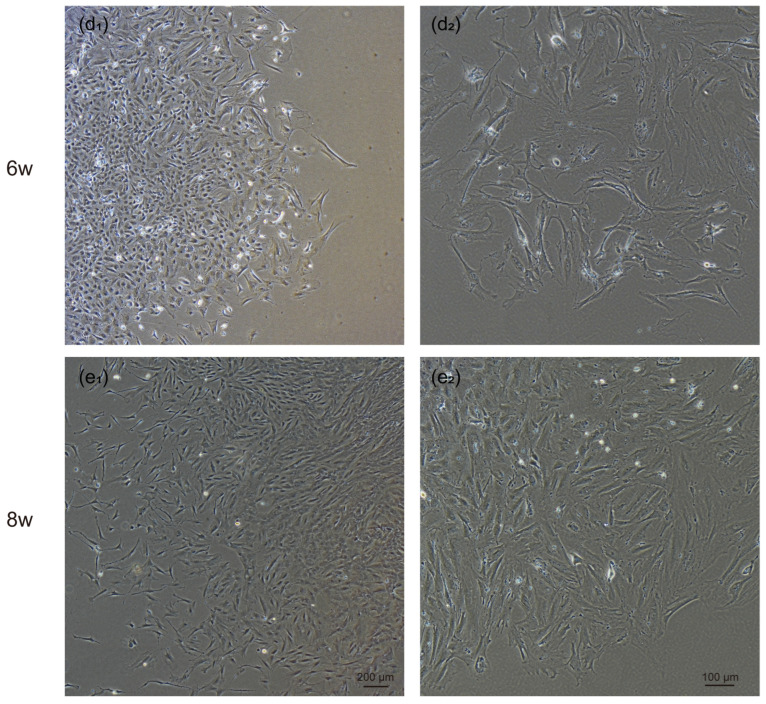
Morphological images of primary cells extracted from the LC tissues of chronic high IOP rats at different time points. Images (**a_1_**), (**a_2_**), (**b_1_**), (**b_2_**), (**c_1_**), (**c_2_**), (**d_1_**), (**d_2_**), (**e_1_**), (**e_2_**) represent primary astrocytes extracted from the control group and from adult rats with chronic high IOP for 2 weeks, 4 weeks, 6 weeks, and 8 weeks, respectively. Images (**a_1_**–**e_1_**) are magnified four times (scale bar: 200 μm), while images (**a_2_**–**e_2_**) show local regions at a magnification of 10 times (scale bar: 100 μm).

**Figure 3:**
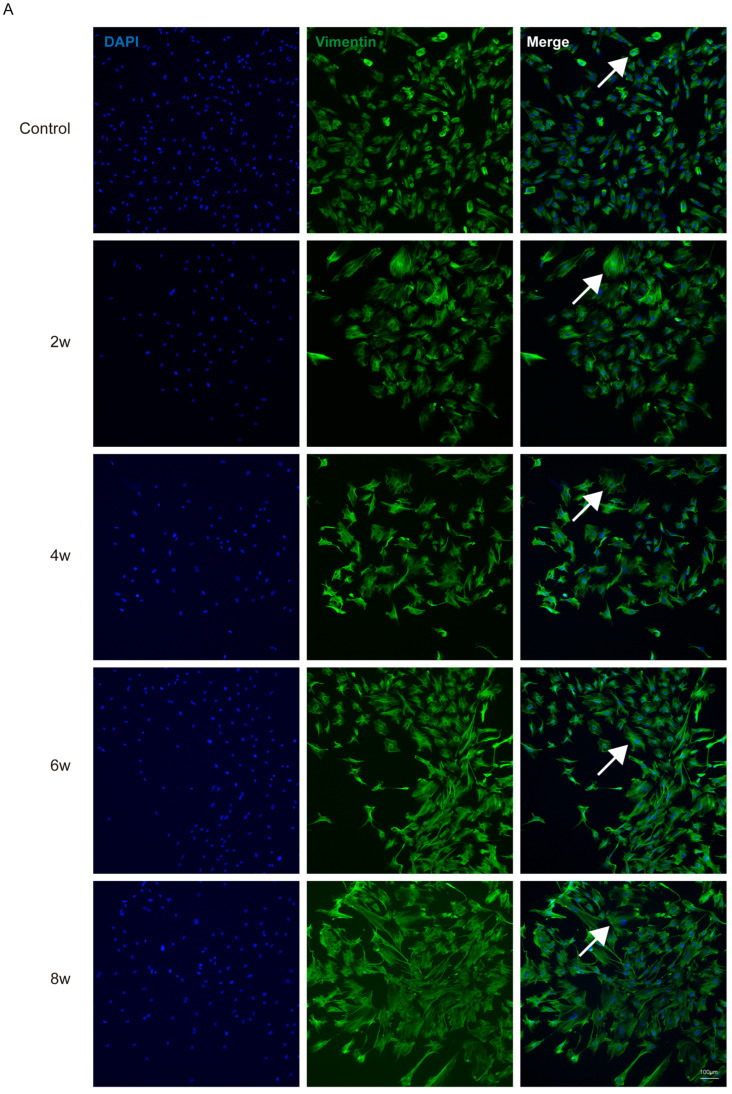

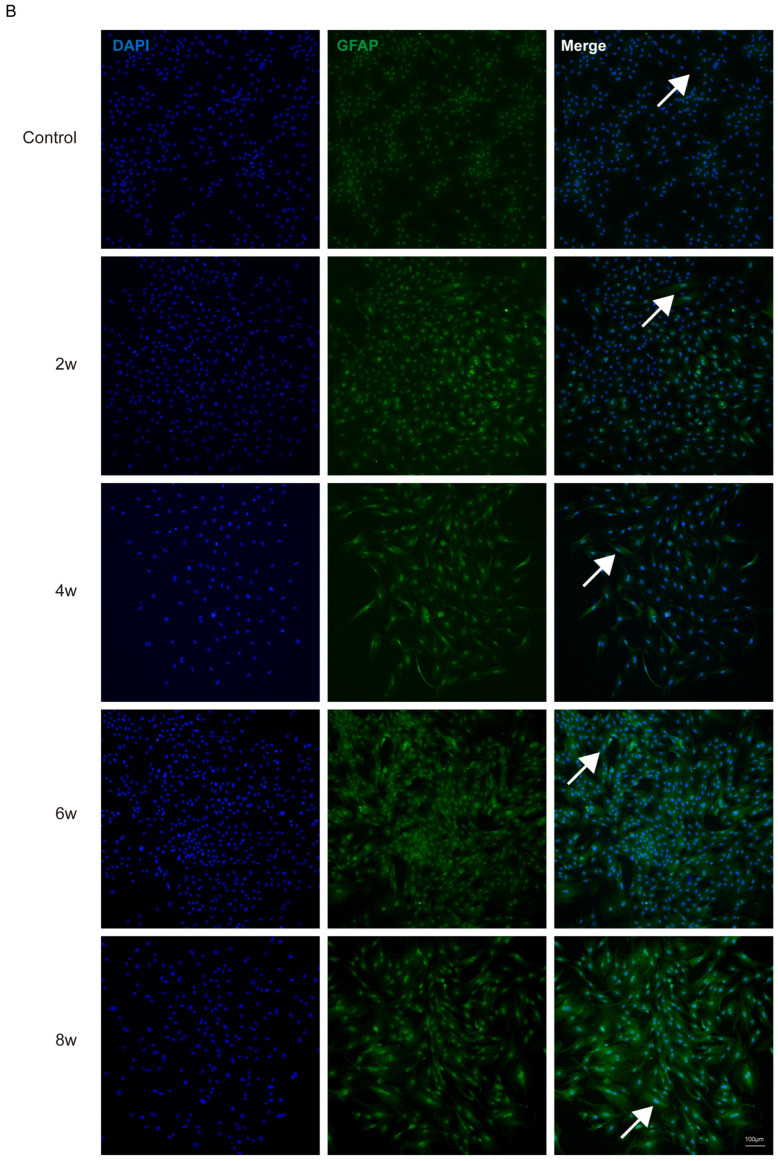

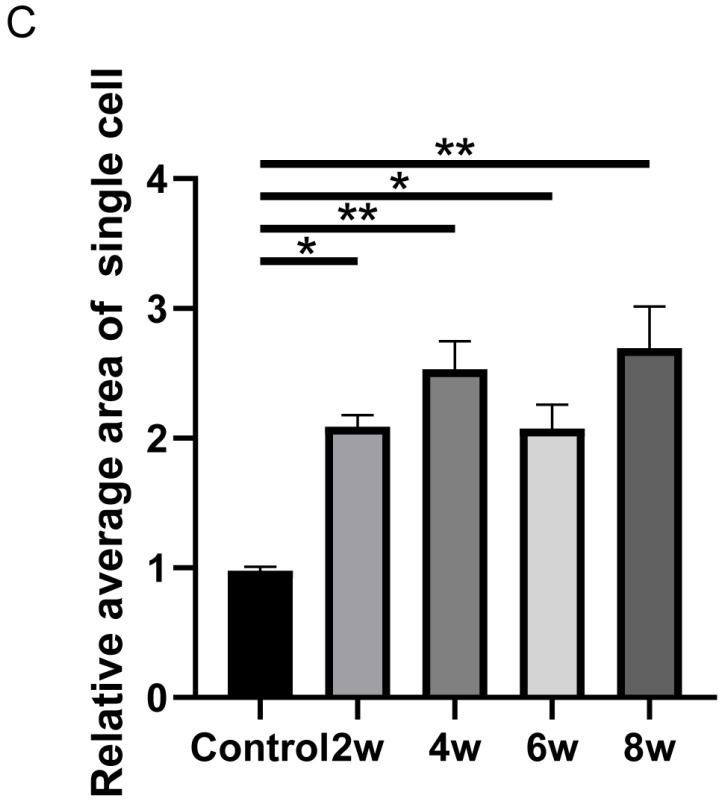
Immunofluorescence staining images of primary astrocytes extracted from the LC tissues of chronic high IOP rats at different time points. (**A**) Vimentin. (**B**) GFAP. The white arrows indicate that astrocytes, after 2, 4, 6, and 8 weeks of elevated IOP, showed a significant increase in cell body size, with more numerous and thicker processes. (**C**) The relative area of astrocytes extracted from the ONH after different durations of high IOP was quantified based on vimentin immunofluorescence staining images. * *p* < 0.05 and ** *p* < 0.01 indicate statistical significance.

**Figure 4:**
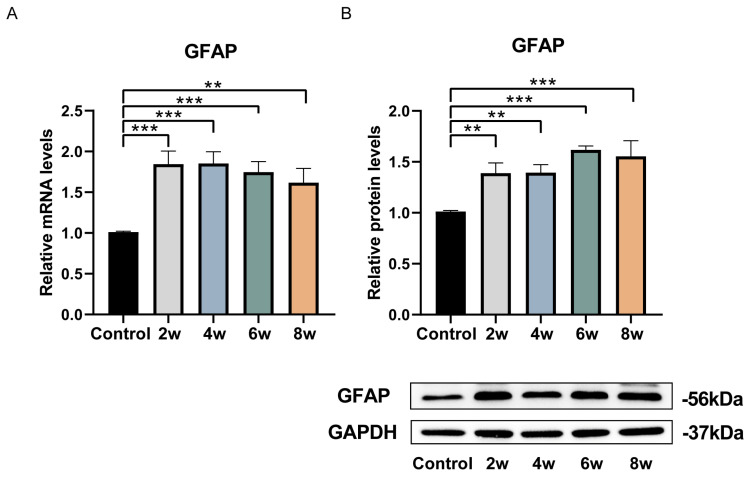
Detection of astrocytes extracted from the LC tissues of chronic high IOP rats at different time points and activation-associated protein GFAP expression by RT-PCR and western blot. (**A**) A statistical analysis chart of relative mRNA expression of GFAP. (**B**) A statistical analysis chart of relative protein expression of GFAP and an immunoblot band image of GFAP protein expression. ** *p* < 0.01 and *** *p* < 0.001 indicate statistical significance.

After approximately 15 days of in vitro adherent culture of rat LC tissue blocks, the cells began to migrate out from the tissue blocks. Following two rounds of purification in the primary glial cell culture medium, the astrocyte-like cell population increased. These cells had abundant cytoplasm, were mostly flat, and exhibited irregular polygonal or star-like shapes with multiple protrusions extending from the cell body (Figure 2). Next, we identified the extracted cells using two astrocyte-specific markers, GFAP and vimentin. The results showed that the extracted cells almost completely expressed the astrocyte-specific markers GFAP (Figure 3A) and vimentin (Figure 3B). Compared to the control group, astrocytes extracted from the LC tissues of rats with elevated IOP after 2, 4, 6, and 8 weeks showed a significant increase in cell body size, with more numerous and thicker processes (Figure 3C). The fluorescence intensity of astrocyte activation-related proteins, GFAP and vimentin, also increased. We further examined the expression of GFAP mRNA and protein in astrocytes extracted from the LC tissues of rats subjected to elevated IOP for varying durations using the qPCR and western blot methods (Figure 4). The findings indicated that, in contrast to the control group, the expression levels of GFAP mRNA (Figure 4A) and protein (Figure 4B) in astrocytes extracted from the LC tissues were significantly increased after 2, 4, 6, and 8 weeks of sustained high IOP. The above results indicate that we successfully extracted astrocytes from the LC tissues of rats subjected to elevated IOP for different durations. The extracted cells were identified to be nearly 100% astrocytes (Table A1). Compared to the control group, astrocytes extracted after 2, 4, 6, and 8 weeks of sustained high IOP showed signs of activation.

**Figure 5:**
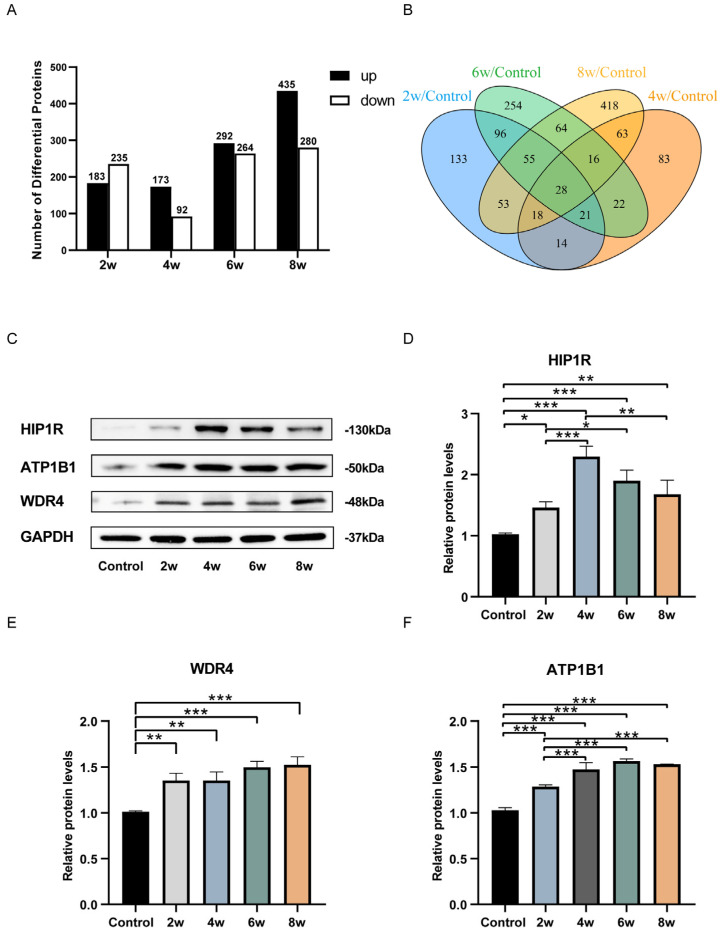
Differentially expressed proteins of astrocytes extracted from the LC tissues of chronic high IOP rats at different time points. (**A**) The number of significantly different proteins. (**B**) Venn diagram of differentially expressed proteins. (**C**) Immunoblot band image of GAPDH, WDR4, ATP1B1, and HIP1R. (**D**–**F**) A statistical analysis chart of relative protein expression of HIP1R (**D**), WDR4 (**E**), and ATP1B1 (**F**). * *p* < 0.05, ** *p* < 0.01, and *** *p* < 0.001 indicate statistical significance.

We performed quantitative analyses of proteins in astrocytes extracted after different durations of elevated IOP using the DIA protein quantification method. Compared to the control group, the differentially expressed proteins in astrocytes extracted from the LC after 2, 4, 6, and 8 weeks of elevated IOP were 418, 265, 556, and 715, respectively (Figure 5A). Among them, the proteins with increased expression were 183, 173, 292, and 435, respectively; the proteins with decreased expression were 235, 92, 264, and 280, respectively. A Venn diagram depicted the intersection of uniquely expressed proteins in astrocytes extracted from the LC tissues after 2, 4, 6, and 8 weeks of sustained high IOP (Figure 5B). Among the differentially expressed proteins, 28 proteins showed significant changes at all four time points (2, 4, 6, and 8 weeks) of elevated intraocular pressure. Detailed information regarding these proteins can be found in Table 1. We selected HIP1R, ATP1B1, and WDR4 to validate the proteomic results, as they are associated with Huntington’s disease, neuronal axon regeneration, and neurological disorders, respectively. HIP1R, ATP1B1, and WDR4 showed significant changes in expression at all four time points of elevated intraocular pressure (Figure 5C–F). Compared to the control group, the protein expression levels of HIP1R, ATP1B1, and WDR4 were significantly increased, a finding which is consistent with the proteomic results.

**Figure 6:**
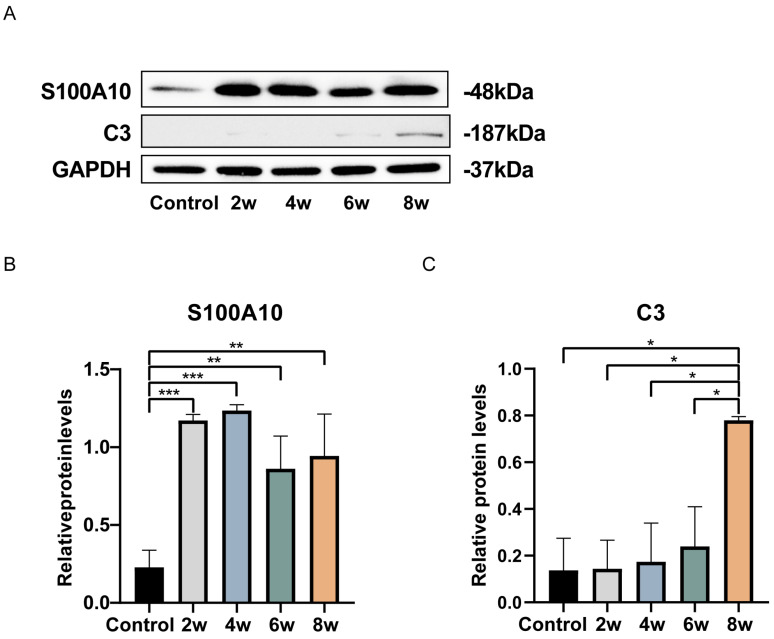
Detection of astrocytes extracted from the LC tissues of chronic high IOP rats at different time points of polarization-associated protein expression by western blot. (**A**) Immunoblot band image of S100A10 and C3 protein expression. (**B**) A statistical analysis chart of the relative protein expression of S100A10. (**C**) A statistical analysis chart of the relative protein expression of C3. * *p* < 0.05, ** *p* < 0.01, and *** *p* < 0.001 indicate statistical significance.

**Figure 7:**
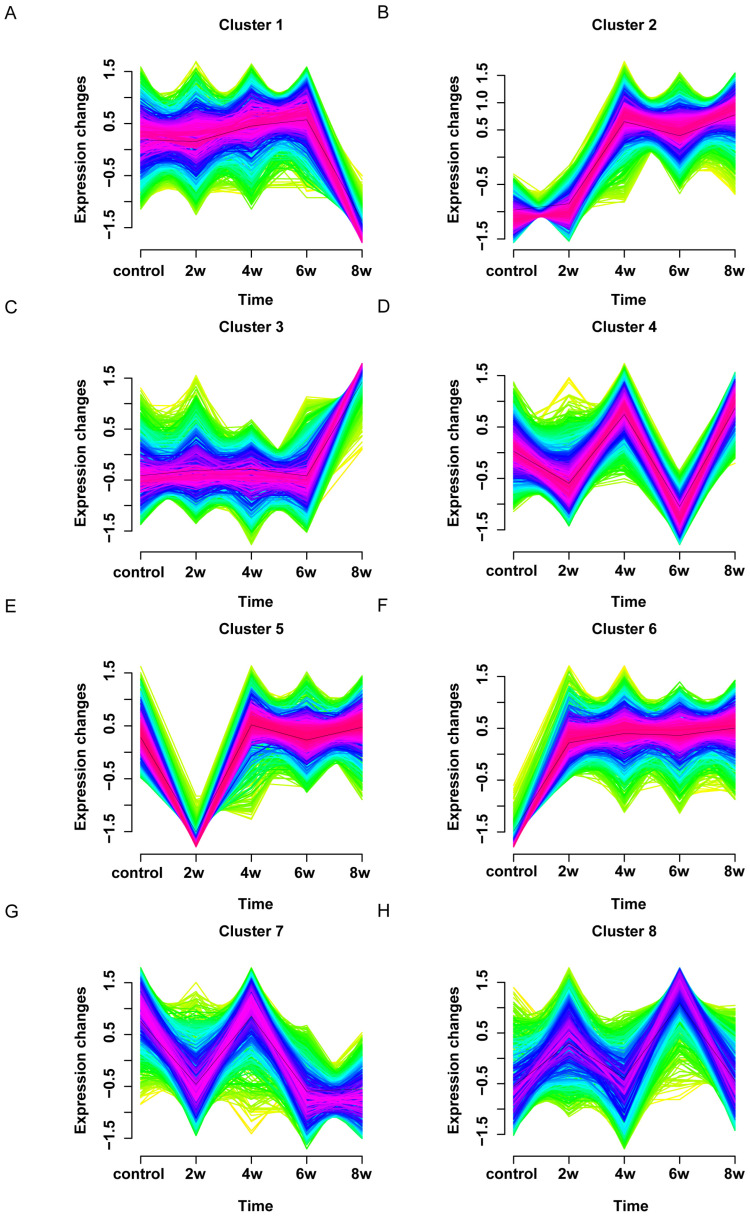
Time-series expression profiles of differential proteins of astrocytes extracted from the LC tissues of chronic high IOP rats at different time points. (**A**–**H**) represent eight different expression patterns of differential proteins.

Next, we examined the expression of proteins related to astrocyte polarization (Figure 6A). Compared to the control group, astrocytes extracted from the LC after 2, 4, 6, and 8 weeks of elevated IOP showed increased expression of S100A10, a specific marker for A2 astrocytes (Figure 6B); astrocytes extracted from the LC after 8 weeks of elevated IOP showed increased expression of C3, a specific marker for A1 astrocytes (Figure 6C). Our results indicate that astrocytes polarize into A2 astrocytes with an increasing duration of elevated IOP. By the 8th week, A1 astrocytes began to appear. To explore the signaling pathways involved in the polarization of A1 and A2 astrocytes, we analyzed the differentially expressed proteins at different time points of elevated IOP in a time-series manner (Figure 7). We divided the differentially expressed proteins into eight clusters based on their expression patterns at different durations of elevated IOP compared to the control group. Among them, the differentially expressed proteins in cluster 3 were highly expressed only after 8 weeks of elevated IOP, similarly to the specific marker C3 of A1 astrocytes (Figure 7C). The differentially expressed proteins in cluster 6 were highly expressed after 2, 4, 6, and 8 weeks of elevated IOP, similarly to the specific marker S100A10 of A2 astrocytes (Figure 7F).

**Figure 8:**
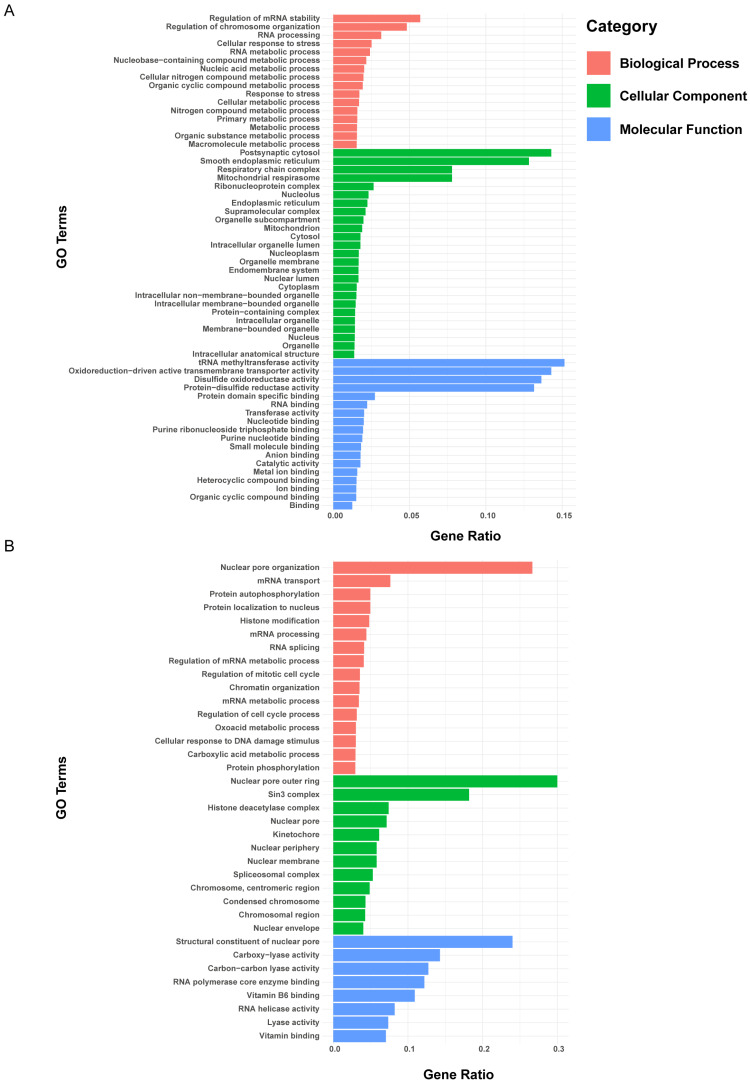
GO enrichment analysis of differential proteins. (**A**) Differential proteins in time-series cluster 3. (**B**) Differential proteins in time-series cluster 6.

**Figure 9:**
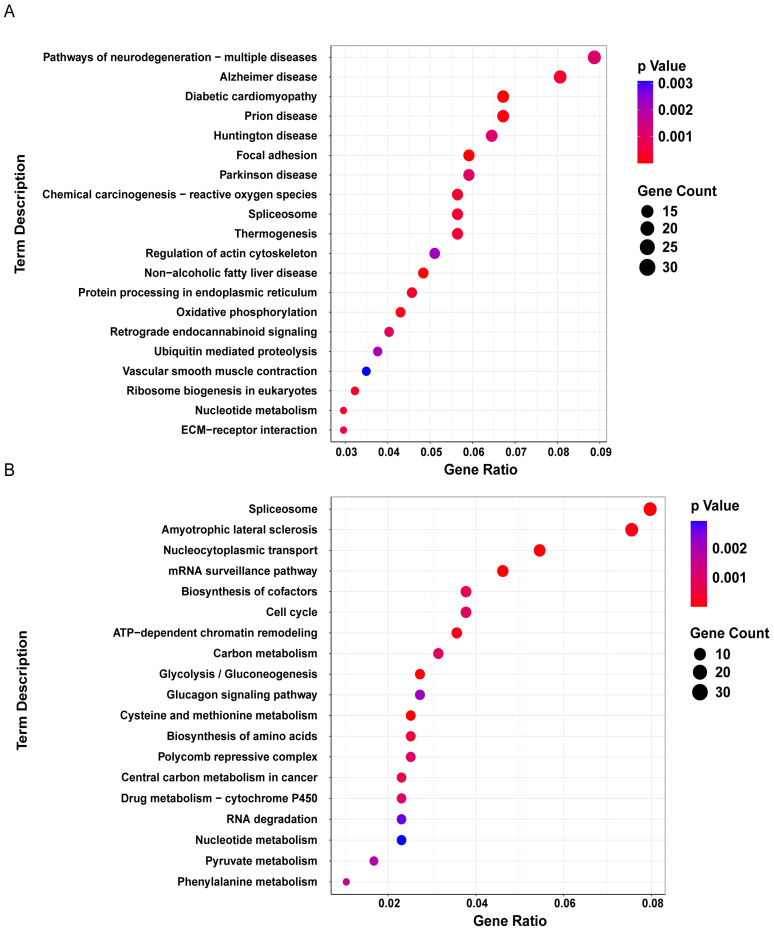
KEGG enrichment analysis of differential proteins. (**A**) Differential proteins in time-series cluster 3. (**B**) Differential proteins in time-series cluster 6.

We performed a Gene Ontology (GO) clustering analysis on the differentially expressed proteins in cluster 3 and cluster 6 (Figure 7C,F). The GO analysis revealed that the differentially expressed proteins in cluster 3 were significantly enriched in biological processes, such as regulation of mRNA stability, regulation of chromosome organization, RNA processing, and cellular response to stress, in cellular components, like postsynaptic cytosol, smooth endoplasmic reticulum, respiratory chain complex, and mitochondrial respirasome, and in molecular functions, including tRNA methyltransferase activity, oxidoreduction-driven active transmembrane transporter activity, disulfide oxidoreductase activity, and protein disulfide reductase activity (Figure 8A). The differentially expressed proteins in cluster 6 were significantly enriched in biological processes, such as nuclear pore organization and mRNA transport, in cellular components, like nuclear pore outer ring and sin3 complex, and in molecular functions, including structural constituent of nuclear pore, carboxy-lyase activity, carbon–carbon lyase activity, RNA polymerase core enzyme binding, and vitamin B6 binding (Figure 8B). These results suggest that the polarization of A1 astrocytes was closely related to astrocyte responses to stress, post-transcriptional regulation, protein translation, and mitochondrial energy metabolism, while the polarization of A2 astrocytes was closely related to changes in nuclear pores. Next, we performed a KEGG enrichment analysis on the differential proteins in cluster 3 and cluster 6 to explore the roles of these proteins, associated with A1 and A2 astrocyte polarization, in metabolism, signaling pathways, and diseases (Figure 9). The results show that the differential proteins associated with A1 astrocyte polarization were primarily enriched in pathways of neurodegeneration with respect to multiple diseases, e.g., Alzheimer’s disease, diabetic cardiomyopathy, prion disease, and Huntington’s disease (Figure 9A). The differential proteins associated with A2 astrocyte polarization were primarily enriched in pathways of spliceosome in amyotrophic lateral sclerosis, nucleocytoplasmic transport, and mRNA surveillance pathway (Figure 9B).

## 4. Discussion and Conclusions

In this study, we established chronic high IOP rat models with durations of 2, 4, 6, and 8 weeks. We extracted astrocytes from the ONH of rats with different durations of high IOP using the tissue block explant method. Astrocytes were activated into reactive astrocytes after 2, 4, 6, and 8 weeks of sustained high IOP. Further research revealed that astrocytes extracted from ONH expressed the A2 astrocyte-specific protein S100A10 under high IOP for 2, 4, 6, and 8 weeks, whereas the expression of the A1 astrocyte-specific protein C3 only began after 8 weeks of high IOP. The differential proteins associated with A1 astrocyte polarization are primarily enriched in pathways of neurodegeneration with respect to multiple diseases, e.g., Alzheimer’s disease, diabetic cardiomyopathy, and Huntington’s disease, while the differential proteins associated with A2 astrocyte polarization are primarily enriched in pathways of spliceosome in amyotrophic lateral sclerosis, nucleocytoplasmic transport, and mRNA surveillance pathway. Our study could provide a better understanding of the role of astrocytes in the ONH for the pathogenesis and progression of glaucoma and offers new perspectives for glaucoma treatment.

Previous research has indicated an increase in the glial LC fraction and astrocyte proliferation during the initial phase of elevated IOP [19]. Numerous studies also indicate that the proliferation of astrocytes and the formation of glial scars play a crucial role in tissue protection and regeneration [12,43,44,45]. During the early stages of glaucoma, the protective role of astrocyte activation predominates [46,47,48]. Widespread suppression of astrocyte reactivity results in poorer visual results and heightened ganglion cell loss after an eye injury [49]. This is consistent with our results, where, in the early stages of elevated IOP, astrocytes in the ONH polarized into the neuroprotective A2 astrocytes. As IOP continued to rise, the neurotoxic A1 astrocytes began to emerge. However, Guttenplan et al.’s study indicated that, in mice with elevated IOP for 4 weeks, the astrocytes in the retina polarized exclusively into A1 astrocytes [50]. The reason might be that retinal astrocytes and ONH astrocytes exhibit heterogeneity [51]. In the ONH of rodents, astrocytes are notably sizable, often spanning at least half the nerve’s width. These cells overlap extensively, with their projections arranged at right angles to the retinal ganglion cell axons, effectively organizing them into distinct bundles [16,52]. Moreover, whether astrocytes take a more neuroprotective or neurotoxic route might be decided not just by the nature of the injury, but also by how close they are, both spatially and temporally, to the site of the injury [53,54,55,56]. This may also explain why ONH astrocytes polarized into A2 astrocytes during the early stages of elevated IOP, while retinal astrocytes polarized into A1 astrocytes.

Previous studies have reported that the dysfunction of astrocytes, through astrogliosis, underlies many neurodegenerative diseases, such as Alexander disease, hepatic encephalopathy, Alzheimer’s disease (AD), Huntington’s disease (HD), Parkinson’s disease (PD), and amyotrophic lateral sclerosis (ALS) [57]. Those studies are consistent with our results. We found that the protein HIP1R, associated with Huntington’s disease, was significantly elevated after 2, 4, 6, and 8 weeks of elevated IOP. Research suggests that GPX3 expression increases in the early stages of AD to alleviate oxidative stress, but its expression decreases in the late stages of AD due to the decompensation of its protective effects [58]. We found that GPX3 expression in astrocytes decreased under elevated IOP, indicating a possible connection between glaucoma and Alzheimer’s disease. Ezrin alleviates the neurotoxicity of α-synuclein in Parkinson’s disease by preventing α-synuclein aggregation [59]. Under elevated IOP, we observed an upregulation of Ezrin expression. These findings suggest a potential link between glaucoma and neurodegenerative diseases. We found that the differential proteins associated with A1 astrocytes are enriched in neurodegeneration-related diseases, e.g., Alzheimer’s disease and Huntington’s disease, while the differential proteins associated with A2 astrocytes are enriched in amyotrophic lateral sclerosis. Our results further illustrate the relationship between neurodegenerative diseases and astrocyte polarization. In addition, a reduction in intron retention in reactive astrocytes has been found in amyotrophic lateral sclerosis [60]. This suggests that the spliceosome plays an important role in astrocyte activation.

There are some limitations in our study. First, A1 astrocytes appeared after 8 weeks of elevated IOP. Therefore, it is necessary to extend the duration of chronic IOP to further investigate the subsequent polarization of astrocytes. Second, since A2 astrocytes appeared in the early stages of elevated intraocular pressure, and A1 astrocytes emerged in the later stages, it remains unclear whether these A1 astrocytes were polarized from resting astrocytes or if they arose from the transformation of A2 astrocytes. Third, to eliminate the influence of other glial cells on the experimental results, the primary astrocytes extracted in this experiment were purified in vitro for 15 days in a medium containing FBS. Studies have revealed that FBS contains substantial amounts of lipopolysaccharides and extracellular vesicles, both of which have been shown to influence cell biology and phenotype [61,62,63]. Therefore, the effect of FBS on astrocytes needs to be considered in subsequent studies. In addition, the specific molecular mechanisms underlying the polarization of A1 and A2 astrocytes remain to be further investigated.

In summary, we investigated the polarization of astrocytes in the ONH under high IOP and the signaling pathways involved in astrocyte polarization. We found that astrocytes in the ONH polarized into the neuroprotective A2 astrocytes after 2, 4, 6, and 8 weeks of elevated IOP, while they polarized into the neurotoxic A1 astrocytes after 8 weeks of elevated IOP. Our research explored astrocyte polarization regulation as a potential glaucoma treatment strategy.

## Figures and Tables

**Table 1:** Common differentially expressed proteins of astrocytes extracted from the LC tissues of chronic high IOP rats at different time points.

Protein_ID	Gene_ID	Fold Change			
		2w	4w	6w	8w
tr_A0A0G2K779_A0A0G2K779_RAT	CEP76	2.83	2.15	2.49	2.55
tr_B5DFK5_B5DFK5_RAT	HIP1R	1.09	1.08	1.11	1.22
tr_A0A0G2K1G5_A0A0G2K1G5_RAT	LTBP2	1.98	1.19	2.04	1.50
sp_Q63531_KS6A1_RAT	RPS6KA1	2.85	1.41	1.48	3.29
tr_D3ZIT4_D3ZIT4_RAT	ANAPC7	1.26	1.31	1.16	1.62
tr_B0BN35_B0BN35_RAT	ELP4	1.32	1.64	1.74	1.89
sp_P31977_EZRI_RAT	EZR	1.09	1.12	1.34	1.01
tr_D3ZY40_D3ZY40_RAT	PCF11	1.79	2.17	1.66	1.95
tr_D4ACM1_D4ACM1_RAT	ELP3	1.15	1.27	1.00	1.82
tr_B2RZB8_B2RZB8_RAT	PSMG4	1.53	1.35	1.31	1.48
tr_D4A0Q3_D4A0Q3_RAT	WDR4	3.01	1.84	2.66	2.18
tr_D4A435_D4A435_RAT	ICAM5	1.95	1.30	2.49	1.82
tr_D4A6W4_D4A6W4_RAT	RFT1	1.12	1.25	1.38	1.20
sp_P07340_AT1B1_RAT	ATP1B1	1.19	1.67	2.27	1.19
sp_O88484_PDP2_RAT	PDP2	2.87	2.29	2.58	2.72
sp_Q6MG60_DDAH2_RAT	DDAH2	−1.07	−1.01	−2.17	−1.01
tr_A0A0G2K0J7_A0A0G2K0J7_RAT	CTDP1	−1.14	−1.39	−1.06	−1.27
sp_Q2YDU8_SPNS1_RAT	SPNS1	−1.25	−1.16	−2.37	−1.28
tr_M0RA39_M0RA39_RAT	MXRA7	−1.40	−1.05	−1.22	−1.25
sp_Q923W4_HDGR3_RAT	HDGFL3	−1.40	−1.11	−1.37	−1.52
sp_P23764_GPX3_RAT	GPX3	−1.46	−1.00	−1.17	−1.39
tr_F1LLW8_F1LLW8_RAT	IDS	−1.53	−1.14	−1.64	−1.16
sp_Q6IRK9_CBPQ_RAT	CPQ	−1.54	−1.54	−1.57	−2.38
sp_P30919_ASPG_RAT	AGA	−1.72	−1.04	−1.78	−2.25
tr_I6LBX8_I6LBX8_RAT	PCDHGC3	−1.90	−1.01	−1.57	−1.14
tr_F1LMV9_F1LMV9_RAT	CORO2B	−2.04	−1.34	−1.63	−2.48
sp_Q6AY80_NQO2_RAT	NQO2	−3.31	−1.71	−4.41	−2.66
tr_Q9JKB7_Q9JKB7_RAT	GDA	−2.04	−2.60	−2.36	1.38

The mean “Fold Change” value derived from three technical replicates is displayed.

## Data Availability

Data are contained within the article.

## Appendix A. The Number of Nuclei and Vimentin-Positive Cells in Vimentin Immunofluorescence Staining of Cells Extracted at Different Time Points Under High IOP

**Table A1:** Count of nuclei and vimentin−positive cells of primary astrocytes extracted from the LC tissues of chronic high IOP rats at different time points.

	Control	2w	4w	6w	8w
The number of cell nuclei	253	86	77	151	132
The number of vimentin−positive cells	253	86	77	151	132
